# Impact of chronic co-infection in pulmonary *Mycobacterium avium* complex disease after treatment initiation

**DOI:** 10.1186/s12890-022-01947-7

**Published:** 2022-04-25

**Authors:** Naohisa Urabe, Susumu Sakamoto, Yui Shimanuki, Takumi Kanokogi, Takumi Motohashi, Nanami Anzai, Chiaki Kato, Asuka Yamaguchi, Nozomi Tokita, Sakae Homma, Kazuma Kishi

**Affiliations:** 1 Department of Respiratory Medicine, Omori Medical Center, Toho University, 6-11-1 Omori-nishi, Ota-ku, Tokyo, 143-8541 Japan; 2 Department of Advanced and Integrated Interstitial Lung Diseases Research, Toho University School of Medicine, Tokyo, Japan

**Keywords:** *Nontuberculous mycobacterium*, *Mycobacterium avium* complex, Co-infection, *Pseudomonas aeruginosa*

## Abstract

**Background:**

The impact of co-infection with other pathogenic microorganisms after initiation of treatment for *Mycobacterium avium* complex pulmonary disease (MAC-PD) has not been clearly described. This study sought to clarify the clinical outcomes of co-infection with MAC after antimycobacterial therapy for MAC.

**Methods:**

Co-infection status was defined as the detection of pathogenic microorganisms other than MAC in at least two consecutive sputum cultures 6–24 months after initiation of treatment. Chest computed tomography (CT) findings and culture results were compared between co-infection and MAC alone groups.

**Results:**

The co-infection and MAC alone groups comprised 12 and 36 patients, respectively. The proportion of patients with sputum culture positive for MAC after 24 months of therapy did not differ significantly between the two groups [25% (3/12) vs. 16.7% (6/36); *p* = 0.671]. The proportion of patients with improved chest CT score after 24 months of starting treatment compared to baseline was significantly lower for the co-infection group than for the MAC alone group [16.7% (2/12) vs. 79.4% (27/34); *p* < 0.001]. In the co-infection group, median CT score values at 12 and 24 months did not differ from baseline. However, the MAC alone group showed significant improvement at 12 and 24 months compared with baseline.

**Conclusions:**

In the patient group with co-infection of other pathogenic microorganisms after treatment initiation for MAC there was no impact on therapeutic efficacy compared to the MAC alone group. However, therapeutic intervention interfered with improvement in chest CT findings such as nodule formation, bronchiectasis, infiltration, and cavitary lesions.

**Supplementary Information:**

The online version contains supplementary material available at 10.1186/s12890-022-01947-7.

## Background

*Nontuberculous mycobacterial *pulmonary disease (NTM-PD) is increasing in incidence worldwide and has become an important concern [1]. Also, the incidence of NTM-PD in Japan is gradually increasing and exceeded the incidence of mycobacterial pulmonary tuberculosis for the first time in 2014 [2]. The type of NTM differs by region. In Japan, *Mycobacterium avium* complex (MAC) accounts for about 90% of the total [2]. In a previous study we reported that 12.2% of patients with MAC pulmonary disease (MAC-PD) had co-infections with *Haemophilus influenzae* (*H. influenzae*) and *Pseudomonas aeruginosa* (*P. aeruginosa*) [3]. According to Fujita et al., 45.1% of patients with MAC-PD had chronic co-infection with other pathogenic microorganisms, and chronic *P. aeruginosa* co-infection increased after the initiation of treatment for MAC-PD [4]. Kamata et al. reported that chronic co-infection with *P. aeruginosa* was seen in 7.8% of patients with MAC-PD [5]. However, the impact of co-infection with other pathogenic microorganisms in MAC-PD after initiation of treatment on the therapeutic efficacy against MAC is not clear.

Thus, we investigated the impact of co-infection with other pathogenic microorganisms after initiation of treatment for MAC-PD.

## Methods

### Study design

This single-center retrospective cohort study included 48 patients with MAC-PD who had started treatment during the period from November 2014 through February 2019 at Toho University Omori Medical Center. All patients were required to fulfill the American Thoracic Society (ATS) criteria for the diagnosis of NTM [6], and they continued MAC-PD treatment from baseline for at least 12 months (46 patients continued for at least 24 months; 2 patients continued for at least 12 months). Sputum culture for mycobacteria, other bacteria, and fungi was performed at least 3 times during the 6–24 months after treatment initiation for MAC-PD. Patients who had difficulty with sputum collection were excluded.

### Definition of co-infection and treatment failure in MAC pulmonary disease

We defined co-infection as the detection of pathogenic microorganisms other than MAC in at least two consecutive sputum cultures 6–24 months after initiation of treatment for MAC-PD. Furthermore, co-infection at baseline was described as ‘base co-infection’. Base co-infection was defined as detection of pathogenic microorganisms other than MAC in at least two separate sputum cultures or at least one bronchoalveolar lavage. The pathogenic microorganisms in ‘co-infection’ are the same pathogens throughout the study, but are different from those in ‘base co-infection’.

Treatment success was defined as sustained negative sputum culture for at least 12 months [7]. Treatment failure was defined as persistent MAC detection in sputum culture after 12 months of treatment.

We compared the clinical characteristics, rate of treatment success, rate of clarithromycin (CAM) resistance, scoring of chest high-resolution CT (HRCT) findings, and subjective symptoms between the co-infection and MAC alone groups. The proportions of patients with improved chest CT and Chronic Obstructive Pulmonary Disease (COPD) assessment testing (CAT) scores at 12 and 24 months after the start of treatment versus baseline were compared between the groups. The CT score and CAT score values at 12 and 24 months were compared with those at baseline for both groups. Univariate and multivariate logistic regression analysis was also performed to identify factors independently associated with co-infection.

### Data collection

The following patient data were collected: age, sex, body mass index (BMI), smoking history, serum anti-glycopeptidolipid-core IgA titer, comorbidities, chest and paranasal sinuses CT, and sputum and bronchoscopy culture results. In total, 43 (90%) patients underwent CAT to confirm subjective symptoms at the time of treatment initiation and at 6, 12, 18, and 24 months thereafter [8].

### Chest CT score

**Fig. 1:**
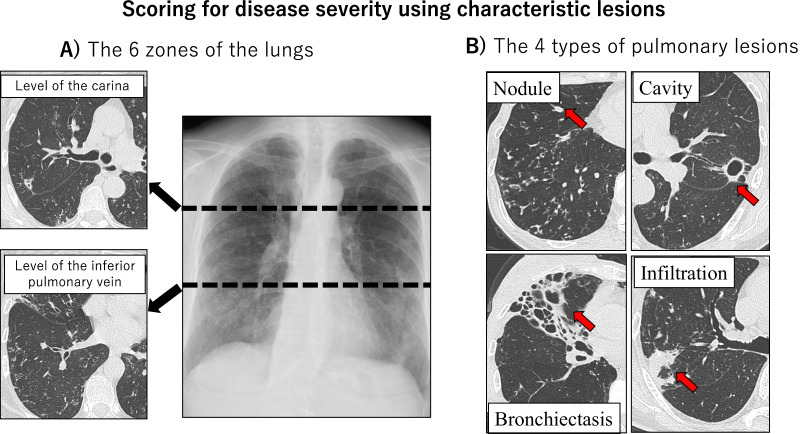
Scoring for disease severity using characteristic lesions. Representative images showing, **A** plain chest radiograph showing the lungs divided into six zones at the levels of the carina and inferior pulmonary vein and **B** the four typical lesions on HRCT described as nodule formation, cavitation, bronchiectasis, and infiltration

**Fig. 2:**
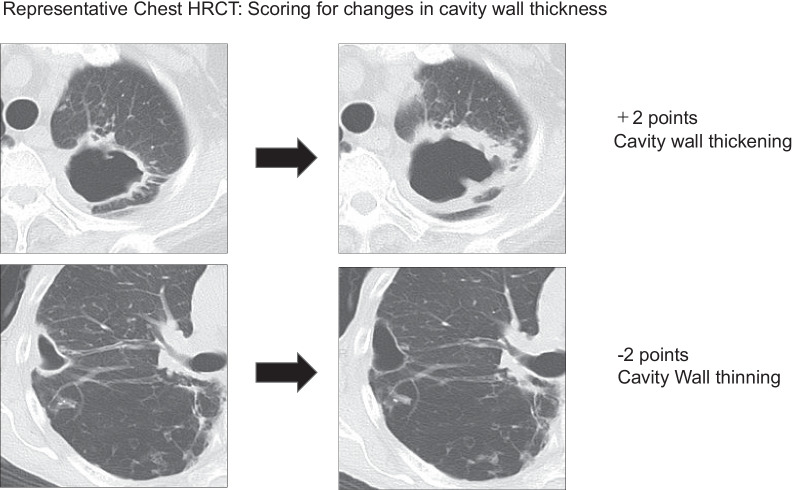
Stages of pulmonary lesions in MAC disease by chest CT score. Cavity wall thickness was assigned + 2 points if the thickness increased by more than 1 mm and − 2 points were given for thinning

Chest HRCT score, taken as an index of disease severity, was reviewed as previously described with slight modification as follows [9]. First, on a plain chest radiograph we divided the lungs into 6 zones using 2 horizontal lines at the levels of the carina and inferior pulmonary vein (Fig. 1). These zones are the right and left upper, middle, and lower zones. Next, using HRCT images, we categorized the 4 characteristic lesions in MAC-PD into 4 types namely, cavities, bronchiectasis, nodules (less than 10 mm), and infiltration (an area of opacity greater than 10 mm)]. A score (from 0 to 4) was assigned to each lesion according to the area occupied by the lesion in each zone that is, 0: no area occupied, 1: 1–24% occupied, 2: 25–49% occupied, 3: 50–74% occupied, and 4: 75–100% occupied as shown in Fig. 1. The sum of the scores for each zone comprised the total score. In addition, for the two largest lung cavities overall, changes in cavity wall thickness were reflected by assigning + 2 points for cavity wall thickening by more than 1 mm and — 2 points for thinning (Fig. 2). The thickness of the cavity wall was determined at the thickest segment of the cavity wall with a measuring system using data from Rapideye Core (Canon Inc., Tokyo, Japan) medical imaging information system. This is an integrated healthcare ICT (medical information) and modality (medical equipment) system for electronic medical records. Two respiratory physicians with over 13 years of experience reviewed Chest CT scores independently. Improved Chest CT score was defined as a decrease of even one point.

### Statistical analysis

Data are presented as the number of patients and percentages. Age, BMI, chest CT score, and CAT score are expressed as median value (with interquartile range). Associations of categorical and continuous variables between patients in the co-infection and MAC alone groups were tested with the chi-squared or Fisher’s exact test, and the Mann–Whitney U test, respectively. Factors independently associated with co-infection were evaluated with univariate and multivariate logistic regression. Multivariate logistic regression was performed using a stepwise method. A *p* value of < 0.05 was considered to indicate statistical significance. Statistical analyses were performed with SPSS software version 22 (IBM Corp., Armonk, NY).

## Results

### Clinical characteristics

In total, 48 patients were included in this study; the co-infection group comprised 12 patients (median age 71.5 years; 2 men, 10 women) and the MAC alone group included 36 patients (median age 71 years; 7 men, 29 women). The characteristics of all 48 patients are shown in Tables 1 and 2. At baseline, there were significant differences between the co-infection and MAC alone groups in scores for chest CT [13 (10.8–14.3) vs. 9 (6.8–11); *p* = 0.007], bronchiectasis lesions score on chest CT [4 (2.8–5) vs. 2 (1–3.3); *p* = 0.035], subjective cough symptoms [2 (2–3) vs. 1 (0–2.3); *p* = 0.015], and sputum [3 (1.5–4) vs. 1 (0–2); *p* = 0.021]. Both groups showed the same resistance rate to CAM of 8.3% at 24 months after treatment initiation. Overall treatment success rate for MAC was 81.2% (39/48) with no significant differences between the co-infection and MAC alone groups (75% vs. 83.3%; *p* = 0.671).

**Table 1:** Clinical characteristics of the MAC alone and co-infection groups

Characteristic	Total	MAC alone group	Co-infection group	*p* value
No. of patients	48	36	12	
Age: (years); median (range)^a^	71 (63.8–78)	71 (62–78)	71.5 (67.8–75.8)	0.195
Gender: female; n (%)	39 (81.3)	29 (80.6)	10 (83.3)	> 0.999
BMI: (kg/m^2^); median (range)^a^	19 (16.3–21)	19 (17.3–21.3)	18.3 (16.2–19.5)	0.301
Smoking: never; n (%)	35 (72.9)	25 (69.4)	10 (83.3)	0.469
Positive result for GPL-core serum IgA; n (%)	38 (79.2)	28 (77.8)	10 (83.3)	> 0.999
Comorbidities; n (%)				
Rheumatoid arthritis (RA)	10 (20.8)	8 (22.2)	2 (16.7)	> 0.999
Sinusitis	4 (8.3)	3 (8.3)	1 (8.3)	> 0.999
Underlying pulmonary disease; n (%)				
Emphysema	6 (12.5)	5 (13.9)	1 (8.3)	> 0.999
Interstitial pneumonia	8 (16.7)	7 (19.4)	1 (8.3)	0.659
Concomitant drug; n (%)				
Corticosteroids	8 (16.7)	6 (16.7)	2 (16.7)	> 0.999
Immunosuppressant	9 (18.8)	7 (19.4)	2 (16.7)	> 0.999
Biopharmaceutical	1 (2.1)	1 (2.8)	0	> 0.999
Infective MAC strain; n (%)				
*M. avium*	36 (75)	29 (80.6)	7 (58.3)	0.143
*M. intracellulare*	16 (33.3)	10 (27.8)	6 (50)	0.178
With sputum; n (%)				
Positive MAC culture	32 (66.6)	25 (69.4)	7 (58.3)	0.500
With bronchoscopy; n (%)				
Positive culture of MAC	35/36 (97.2)	27/28 (96.4)	6/6 (100)	> 0.999
Co-infection at baseline; n(%)	20 (41.7)	14(38.9)	6 (50)	0.520
With sputum	10 (20.8)	6 (16.7)	4 (33.3)	0.241
With bronchoscopy	14/33 (42.4)	11/28 (39.3)	3/6 (50)	0.672
CAM resistant strain; n (%)				
At baseline	2 (4.2)	1 (2.8)	1 (8.3)	0.441
24 months after treatment initiation	4 (8.3)	3 (8.3)	1 (8.3)	> 0.999
Treatment for MAC; n (%)				
CAM/EB/RFP	39 (81.3)	29 (80.6)	10 (83.3)	> 0.999
CAM/EB	6 (12.5)	6 (16.7)	0	0.315
Other	3 (6.3)	1 (2.8)	2 (16.7)	0.156
Treatment success; n (%)	39 (81.2)	30 (83.3)	9 (75)	0.671

MAC, *Mycobacterium avium* complex; BMI, body mass index; GPL, glycopeptidolipid; CAM, clarithromycin; EB, ethambutol; RFP, rifampicin

^a^Interquartile range

**Table 2:** Imaging findings and subjective symptoms of the MAC alone and co-infection groups

Characteristic	Total	MAC alone group	Co-infection group	*p* value
No. of patients	48	36	12	
Chest CT imaging results				
Chest CT score; median (range)^a^	10 (7–13)	9 (6.8–11)	13 (10.8–14.3)	0.007
Bronchiectasis lesion score in chest CT score	2.5 (1–4)	2 (1–3.3)	4 (2.8–5)	0.035
Cavity lesion score in chest CT score	0 (0–1)	1 (0–1)	0 (0–0.3)	0.117
Cavitary lesion; n (%)	21 (43.8)	18 (50)	3 (25)	0.185
Radiographic pattern; n (%)				
Noncavitary NBE type	27 (56.3)	18 (50)	9 (75)	0.315
FC type	6 (12.5)	6 (16.7)	0	0.315
Cavitary NBE type	15 (31.3)	12 (33.3)	3 (25)	0.728
Subjective symptoms				
CAT score	6 (4.5–12.5)	6 (4–10.5)	9 (6–15)	0.211
Cough + Sputum score^b^	2 (1–5)	2 (1–4)	6 (3.5–6.5)	0.005
Cough score^b^	1 (1–3)	1 (0–2.3)	2 (2–3)	0.015
Sputum score^b^	1 (0.5–2)	1 (0–2)	3 (1.5–4)	0.021
Chest tightness^b^	0 (0–1)	0 (0–1)	1 (0–2)	0.386
Breathlessness^b^	1 (0–2.5)	1.5 (0.8–3)	0 (0–2)	0.393
Limited activity^b^	0 (0–0)	0 (0–0)	0 (0–0)	0.681
Confidence leaving home^b^	0 (0–1)	0 (0–1)	0 (0–2)	0.347
Sleeplessness^b^	0 (0–1)	0 (0–1)	0 (0–1.5)	0.862
Energy^b^	1 (0–2)	1 (0–2)	1 (0.5–2)	0.924

MAC, *Mycobacterium avium* complex; CT, computed tomography; NBE, nodular bronchiectatic; FC, fibrocavitary; CAT, COPD assessment test

^a^Interquartile range

^b^Score for subjective symptoms of cough and sputum, included in CAT score

### Microbiological test results in the co-infection group

Serial changes in microbiological test results in the co-infection group are shown in Table 3. *P. aeruginosa* was the most common pathogenic microorganism in co-infections after the treatment for MAC and was detected in 7 of 12 (58.3%) patients. *Nocardia* spp. was detected in 2 of 12 (16.7%) patients, and *Methicillin-sensitive Staphylococcus aureus* (MSSA), *Escherichia coli* (*E. coli*) and *Serratia marcescens* were detected in 1 (8.3%) patient each. The Geckler classification of sputum examination is shown in Additional file 1: Table S1. The pathogenic microorganism was detected in 10 of the 12 patients in the co-infection group, at least once from examination of high-quality sputum samples (Geckler classification 4 or 5) [10].

**Table 3:** Sputum culture changes in the co-infection group

No	Baseline					6–12 months			12–24 months		
	Bronchoscopy		Sputum			Sputum			Sputum		
	Mycobacteria	Bacteria	Count	Mycobacteria	Bacteria	Count	Mycobacteria	Bacteria	Count	Mycobacteria	Bacteria
1			3	*M. intracellulare*	*E. coli*^6^ × 2	6	Negative	Negative	6	*M. intracellulare*	*E. coli* × *2*
2	*M. avium*^1^	MSSA^3^	6	Negative	MSSA × 1	3	Negative	Negative	2	Negative	*P. aeruginosa* × 2
3	*M. avium/intracellulare*^2^	*P. aeruginosa*^4^	2	Negative	Negative	1	Negative	Negative	3	Negative	*P. aeruginosa* × 3
4			3	*M. intracellulare*	MSSA × 3	3	Negative	MSSA × 2	4	Negative	MSSA × 3, *H. influenzae* × 1
5			8	*M. intracellulare*	*H. influenzae* × 2	3	Negative	Negative	3	Negative	*P. aeruginosa* × 2
6	*M. avium*	*H. influenzae*^5^	3	Negative	*H. influenzae* × 2 *P. aeruginosa* × 1	2	Negative	Negative	3	Negative	*P. aeruginosa* × 2
7	*M. intracellulare*		3	*M. intracellulare*	Negative	5	Negative	*P. aeruginosa* × 5	5	Negative	*P. aeruginosa* × 5
8	*M. avium*		2	Negative	Negative	1	Negative	Negative	3	Negative	*Nocardia* spp. × 2
9			3	*M. intracellulare*	Negative	1	*M. intracellulare*	Negative	4	*M. avium*	*Nocardia* spp. × 2
10			2	*M. avium*	Negative	1	Negative	Negative	3	Negative	*P. aeruginosa* × 3
11	*M. avium*		3	Negative	Negative	2	Negative	Negative	2	Negative	*P. aeruginosa* × 2
12			3	*M. avium*	Negative	2	*M. avium*	*S. marcescens*^7^ × 2	3	*M. avium*	*S. marcescens* × 2

1, *Mycobacterium avium*; 2, *Mycobacterium intracellulare*; 3, *Methicillin-sensitive Staphylococcus aureus*; 4, *Pseudomonas aeruginosa*; 5, *Haemophilus influenzae*; 6, *Escherichia coli*; 7, *Serratia marcescens*

### Serial changes in Chest CT score

**Fig. 3:**
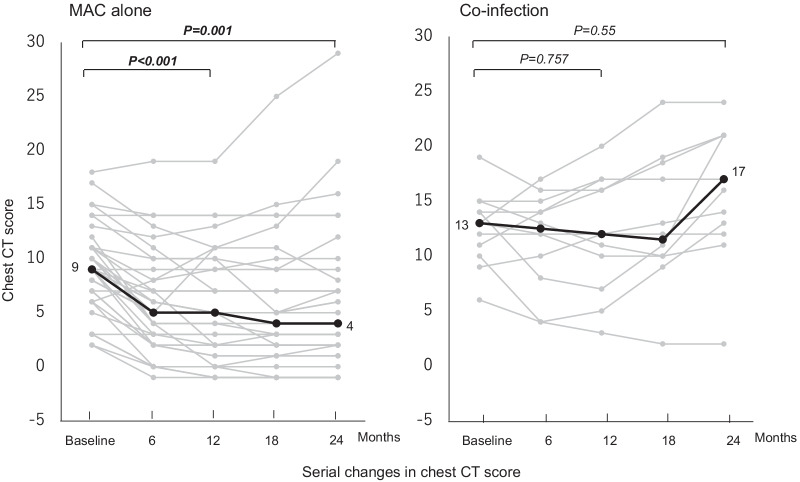
Serial changes in chest CT score. Gray lines indicate change in chest CT score in each patient after initiation of treatment for pulmonary MAC disease. Black lines indicate the median for all patients. A paired t-test was used to compare values at 12 and 24 months with baseline

Table 4 shows the clinical course in patients with MAC-PD. The CT score, as a measure of disease severity, was improved in 72.9% and 63% of patients at 12 months and 24 months after treatment initiation. At 24 months after treatment, the proportion of patients with improved chest CT score was significantly lower in the co-infection group than the MAC alone group (16.7% vs. 79.4%; *p* < 0.001). Figure 3 shows serial changes in chest CT score in the 2 groups. In the co-infection group, mean CT score at 12 and 24 months did not differ compared with the baseline CT score [12(9.3–16.3) and 17(13–21) vs. 13(10.8–14.3); *p* = 0.757 and *p* = 0.55]. However, in the MAC alone group, mean CT score at 12 and 24 months was significantly improved compared with the baseline score [5(2–9.3) and 4(2–7.8) vs. 9(6.8–11); *p* < 0.001 and *p* < 0.001]. The Chest CT score for all patients is shown in Additional file 2: Table S2.

**Table 4:** Comparison of clinical course of pulmonary MAC patients after treatment initiation

Proportion of patients, Parameter	Total	MAC alone group	Co-infection group	*P* value
Improved CT score, % (n)				
12 months after treatment initiation	72.9% (35/48)	80.6% (29/36)	50% (6/12)	0.061
24 months after treatment initiation	63% (29/46)	79.4% (27/34)	16.7% (2/12)	< 0.001
Improved CAT score, % (n)				
12 months after treatment initiation	58.5% (24/41)	54.8% (17/31)	70% (7/10)	0.48
24 months after treatment initiation	71.8% (28/39)	48.3% (14/29)	40% (4/10)	0.726

MAC, *Mycobacterium avium* complex; CAT, COPD assessment test

Score for subjective symptoms of cough and sputum, included in CAT score

### Serial changes in CAT score

**Fig. 4:**
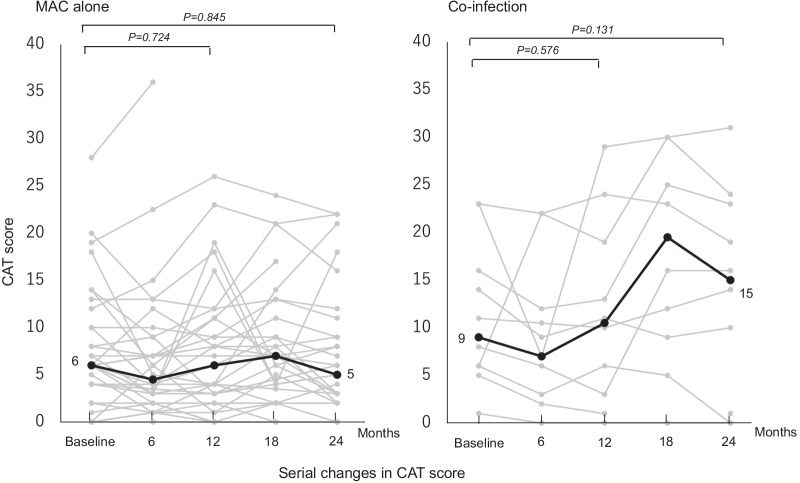
Serial changes in CAT score. Gray lines indicate change in CAT score in each patient after initiation of treatment for MAC pulmonary disease. Black lines indicate the median for all patients. A paired t-test was used to compare values at 12 and 24 months with baseline values

In a comparison of subjective symptoms between the two groups, at 12 and 24 months after initiation of treatment for MAC-PD the proportion of patients with improved CAT scores did not differ significantly between the co-infection and MAC alone groups (70% vs. 54.8% and 40% vs. 48.3%; *p* = 0.48 and *p* = 0.726) (Table 4). Figure 4 shows serial changes in CAT score. The co-infection group showed no significant difference in mean CAT score at 12 and 24 months compared with baseline [10.5(3.8–17.5) and 15(3.3–22) vs. 9(6–15); *p* = 0.576 and *p* = 0.131]; in the MAC alone group the CAT score at 12 and 24 months did not differ significantly from the baseline score [6(2.5–11) and 5(2–9) vs. 6(4–10.5); *p* = 0.724 and *p* = 0.845].

### Risk factors for co-infection

Table 5 shows the results of univariate and multivariate logistic regression analysis of independent associations with co-infection. Higher chest CT score (Odds Ratio [OR], 1.27; 95% confidence interval [CI], 1.04–1.55; *p* = 0.017), higher sputum score (OR, 2.35; 95% CI, 1.28–4.31; *p* = 0.006), and higher cough score (OR, 2.2; 95% CI, 1.17–4.14; *p* = 0.014) were independently associated with co-infection in univariate analysis. Higher sputum score (OR, 2.02; 95% CI, 1.08–3.79; *p* = 0.028) was independently associated with co-infection in multivariate analysis.

**Table 5:** Univariate and multivariate logistic regression analysis of independent associations with co-infection

Variable	Univariate logistic regression			Multivariate logistic regression		
	OR	95% CI	*p* value	OR	95% CI	*p* value
Chest CT score	1.27	1.04–1.55	0.017	1.18	0.95–1.47	0.138
Age (years)	1.04	0.967–1.11	0.308			
BMI	0.90	0.72–1.13	0.356			
*M. intracellulare*^a^	2.60	0.68–9.99	0.164			
CAT score	1.07	0.97–1.18	0.184			
Sputum score^b^	2.35	1.28–4.31	0.006	2.02	1.08–3.79	0.028
Cough score^b^	2.2	1.17–4.14	0.014	1.51	0.65–3.52	0.340

OR, odds ratio; CI, confidence interval; BMI, body mass index; *M. intracellulare, Mycobacterium intracellulare*; CAT, COPD assessment test

^a^Odds ratio versus M. avium

^b^Score for subjective symptoms of cough and sputum, included in CAT score

## Discussion

We demonstrated the impact of co-infection with other pathogenic microorganisms after initiation of treatment for MAC-PD. Although co-infection with other pathogenic microorganisms does not affect therapeutic efficacy against MAC, these organisms may interfere with improvement of chest CT findings. Few studies have investigated the impact of co-infection with other pathogenic microorganisms in MAC-PD [4, 5]. It is important to note that these were cross-sectional studies, and so did not clarify the impact of co-infection on the efficacy of MAC treatment. To our knowledge, this is the first report to investigate the impact of co-infection with other pathogenic microorganisms on clinical course after initiation of treatment for MAC-PD.

This study showed that treatment success rate was 81%, improvement in chest CT score was 63%, and the rate of CAM resistance was 8.3%. These results were consistent with those from previous studies. Earlier reports of macrolide-inclusive daily regimens have shown that the rate of sputum culture conversion was 42–92% [11–15], chest imaging improvement was 68–82% [13, 14], and macrolide resistance was 9–15% [11, 12, 15]. In addition, the rates of MAC culture conversion and CAM resistance did not differ significantly between the co-infection and MAC alone groups. These results suggest that co-infection after the initiation of treatment for MAC-PD did not affect the treatment efficacy.

In the co-infection group, CAM-susceptible bacteria such as MSSA and *H. influenzae* decreased after MAC treatment while CAM-resistant bacteria such as *P. aeruginosa* and *Nocardia* spp. increased after MAC treatment. We speculated that MAC treatment suppressed the proliferation of MAC and other CAM-susceptible bacteria, and this might foster a conversion of the bacteria to CAM-resistant. This result is consistent with previous reports showing that *P. aeruginosa* was less frequently isolated from positive MAC sputum cultures and more often isolated after MAC sputum conversion [4]. The worse chest CT findings may have been as a result of other pathogenic microorganisms that gained dominance due to weakening of the competing MAC. Also, in the MAC alone group the disappearance of “base co-infection” over time could be because this was contamination. Another possibility is that microorganisms may have been eradicated by treatment with CAM or rifampicin for MAC.

Previous studies showed that NTM-PD including those with cystic fibrosis, had a lower rate of chronic *P. aeruginosa* infection compared with non-NTM infection [16, 17]. MAC and other pathogenic microorganisms, especially *P. aeruginosa*, interact with each other and culture results may reflect the dominant pathogenic species at that time. Therefore, we speculate that the negative MAC culture in the co-infection group may not only be due to the effect of MAC treatment but also due to the suppression of MAC culture by other potentially infectious pathogenic microorganisms that became dominant.

In this study, subjective symptoms were more severe in the co-infection group at baseline. Specifically, high sputum score at baseline was an independent risk factor for co-infection. According to Kamata et al., co-infection with *P. aeruginosa* worsened subjective symptoms in patients with MAC-PD [4]. Previous reports showed that *P. aeruginosa* colonization was an independent predictor of hospital admission in bronchiectasis [18]. But in our study, the rate of “base co-infection” showed no significant difference between the co-infection and MAC alone groups. We surmised that the presence of trace amounts of other bacteria undetectable by conventional culture in the co-infection group at baseline was the cause of the severe subjective symptoms, and that the bacteria may have become apparently detectable with MAC treatment.

Several risk factors for developing co-infection in patients with MAC-PD have been reported. In the Fujita study, risk factors for co-infection were reported to be COPD and *M. intracellulare* infection [4]. Their study included 18 of 124 patients who had aspergillus co-infection. In contrast, the Kamata study included 19 patients with *P. aeruginosa* infection only, and the severity of bronchiectasis, not cavitary lesions, was associated with *P. aeruginosa* co-infection. The disparity in risk factors for co-infection in these studies may be due to the presence or absence of *Aspergillus*. Cavitary lesions have been reported to be a risk factor for complications of chronic pulmonary aspergillus infection [19] and *M. intracellulare* infection was more likely to show fibrocavitary disease than *M. avium* infection [20]. In addition, the severity of bronchiectasis was significantly associated with the presence of chronic *P. aeruginosa* infection in patients with non-cystic fibrosis bronchiectasis [21]. In this study, 7 out of 12 patients had *P. aeruginosa* co-infection and none had *Aspergillus* co-infection. Also, bronchiectasis score was significantly higher in the co-infection group than the MAC alone group. However, cavitary lesion score and the frequency of concomitant emphysema did not differ between the two groups.

This study revealed considerable discrepancy between radiographic and symptomatic improvement. We considered that CAT score might not reflect the disease status of MAC-PD over time because it does not include other MAC-specific symptoms such as hemoptysis, weight loss, anorexia, and low-grade fever. It is also possible that there was no accurate association due to the small number of cases. Further study is thus needed.

These findings notwithstanding, this study had several limitations that should be mentioned. Firstly, it was a single-center study in a small number of patients. Thus, the findings may not be generalizable to a larger, more diverse population. Secondly, some patients were excluded from this study due to missing sputum examination and chest CT evaluations. These excluded patients might have had infections from a different type of pathogen. Thirdly, the potential presence of indigenous oral bacterial populations cannot be ruled out, and so this may not accurately reflect the status of the lower airway flora because not all patients underwent bronchoscopy. Fourth, since patients were selected based on identified variables (co-infection), there might be some degree of selection bias in the analysis of risk factors for co-infection.

## Conclusion

In the patient group with co-infection of other pathogenic microorganisms after treatment initiation for MAC-PD there was no impact on therapeutic efficacy compared to the MAC alone group. However, therapeutic intervention affected improvement in chest CT findings such as nodule formation, bronchiectasis, infiltration, and cavitary lesions.

## Supplementary Information

**Additional file 1: Supplementary Table 1.** Detailed sputum culture changes of all patients.

**Additional file 2: Supplementary Table 2.** Detailed CT scoring changes of all patients.

## Data Availability

All data generated or analysed during this study are included in this published article [and its additional files].

## Abbreviations

ATS: American Thoracic Society
BMI: Body mass index
COPD: Chronic Obstructive Pulmonary Disease
CAT: Chronic Obstructive Pulmonary Disease assessment testing
CAM: Clarithromycin
CT: Computed tomography
CI: Confidence interval
*E. coli*: *Escherichia coli*
*H. influenzae*: *Haemophilus influenzae*
HRCT: High-resolution CT
MSSA: *Methicillin-sensitive Staphylococcus aureus*
MAC: *Mycobacterium avium* Complex
MAC-PD: Pulmonary *Mycobacterium avium* complex disease
NTM: *Nontuberculous mycobacteria*
NTM-PD: Pulmonary n*ontuberculous mycobacteria* disease
OR: Odds ratio
*P. aeruginosa*: *Pseudomonas aeruginosa*

## References

[1] Brode SK, Daley CL, Marras TK (2014). The epidemiologic relationship between tuberculosis and non-tuberculous mycobacterial disease: a systematic review. Int J Tuberc Lung Dis.

[2] Namkoong H, Kurashima A, Morimoto K, Hoshino Y, Hasegawa N, Ato M (2016). Epidemiology of pulmonary nontuberculous mycobacterial disease. Jpn Emerg Infect Dis.

[3] Urabe N, Sakamoto S, Sano G, Ito A, Homma S (2018). Characteristics of patients with bronchoscopy-diagnosed pulmonary Mycobacterium avium complex infection. J Infect Chemother.

[4] Fujita K, Ito Y, Hirai T, Kubo T, Togashi K, Ichiyama S (2014). Prevalence and risk factors for chronic co-infection in pulmonary Mycobacterium avium complex disease. BMJ Open Respir Res.

[5] Kamata H, Asakura T, Suzuki S, Namkoong H, Yagi K, Funatsu Y (2017). Impact of chronic *Pseudomonas aeruginosa* infection on health-related quality of life in Mycobacterium avium complex lung disease. BMC Pulm Med.

[6] Griffith DE, Aksamit T, Brown-Elliott BA, Catanzaro A, Daley C, Gordin F (2007). An official ATS/IDSA statement: diagnosis, treatment, and prevention of nontuberculous mycobacterial diseases. Am J Respir Crit Care Med.

[7] Van Ingen J, Aksamit T, Andrejak C, Böttger EC, Cambau E, Daley CL (2018). Treatment outcome definitions in nontuberculous mycobacterial pulmonary disease: an NTM-NET consensus statement. Eur Respir J.

[8] Jones PW, Tabberer M, Chen WH (2011). Creating scenarios of the impact of COPD and their relationship to COPD Assessment Test (CAT™) scores. BMC Pulm Med.

[9] Morimoto K, Yoshiyama T, Kurashima A, Sasaki Y, Hoshino Y, Yoshimori K (2014). Nutritional indicators are correlated with the radiological severity score in patients with Mycobacterium avium complex pulmonary disease: a cross-sectional study. Intern Med.

[10] Geckler RW, Gremillion DH, McAllister CK, Ellenbogen C (1977). Microscopic and bacteriological comparison of paired sputa and transtracheal aspirates. J Clin Microbiol.

[11] Wallace RJ, Brown BA, Griffith DE, Girard WM, Murphy DT (1996). Clarithromycin regimens for pulmonary Mycobacterium avium complex. The first 50 patients. Am J Respir Crit Care Med.

[12] Griffith DE, Brown BA, Cegielski P, Murphy DT, Wallace RJ (2000). Early results (at 6 months) with intermittent clarithromycin-including regimens for lung disease due to Mycobacterium avium complex. Clin Infect Dis.

[13] Jeong BH, Jeon K, Park HY, Kim SY, Lee KS, Huh HJ (2015). Intermittent antibiotic therapy for nodular bronchiectatic Mycobacterium avium complex lung disease. Am J Respir Crit Care Med.

[14] Miwa S, Shirai M, Toyoshima M, Shirai T, Yasuda K, Yokomura K (2014). Efficacy of clarithromycin and ethambutol for Mycobacterium avium complex pulmonary disease. A preliminary study. Ann Am Thorac Soc.

[15] Ito Y, Miwa S, Shirai M, Kanai M, Fujita K, Ohba H (2020). Macrolide resistant Mycobacterium avium complex pulmonary disease following clarithromycin and ethambutol combination therapy. Respir Med.

[16] Binder AM, Adjemian J, Olivier KN, Prevots DR (2013). Epidemiology of nontuberculous mycobacterial infections and associated chronic macrolide use among persons with cystic fibrosis. Am J Respir Crit Care Med.

[17] Aksamit TR, O'Donnell AE, Barker A, Olivier KN, Winthrop KL, Daniels MLA (2017). Adult patients with bronchiectasis: a first look at the US bronchiectasis research registry. Chest.

[18] Chalmers JD, Goeminne P, Aliberti S, McDonnell MJ, Lonni S, Davidson J (2014). The bronchiectasis severity index. An international derivation and validation study. Am J Respir Crit Care Med.

[19] Song JW, Koh WJ, Lee KS, Lee JY, Chung MJ, Kim TS (2008). High-resolution CT findings of Mycobacterium avium-intracellulare complex pulmonary disease: correlation with pulmonary function test results. AJR Am J Roentgenol.

[20] Koh WJ, Jeong BH, Jeon K, Lee NY, Lee KS, Woo SY (2012). Clinical significance of the differentiation between Mycobacterium avium and Mycobacterium intracellulare in M. avium complex lung disease. Chest.

[21] Dimakou K, Triantafillidou C, Toumbis M, Tsikritsaki K, Malagari K, Bakakos P (2016). Non CF-bronchiectasis: aetiologic approach, clinical, radiological, microbiological and functional profile in 277 patients. Respir Med.

